# Mild traumatic brain injury recovery: a growth curve modelling analysis over 2 years

**DOI:** 10.1007/s00415-020-09979-x

**Published:** 2020-06-13

**Authors:** Ellen L. Carroll, Joanne G. Outtrim, Faye Forsyth, Anne E. Manktelow, Peter J. A. Hutchinson, Olli Tenovuo, Jussi P. Posti, Lindsay Wilson, Barbara J. Sahakian, David K. Menon, Virginia F. J. Newcombe

**Affiliations:** 1 Division of Anaesthesia, Department of Medicine, University of Cambridge, Cambridge, UK; 2 Division of Neurosurgery, Department of Clinical Neurosciences, University of Cambridge, Cambridge, UK; 3 Turku Brain Injury Centre, Turku University Hospital, Turku, Finland; 4 Department of Clinical Neurosciences, University of Turku, Turku, Finland; 5 Department of Neurosurgery, Neurocenter, Turku University Hospital, Turku, Finland; 6 Division of Psychology, University of Stirling, Stirling, UK; 7 Department of Psychiatry, University of Cambridge, Cambridge, UK; 8 Behavioural and Clinical Neuroscience Institute, University of Cambridge, Cambridge, UK; 9 Department of Clinical Neurosciences, University of Cambridge, Cambridge, UK; 10 Wolfson Brain Imaging Centre, University of Cambridge, Cambridge, UK

**Keywords:** Mild traumatic brain injury, Latent growth curve modelling, Cognitive impairment, Outcome

## Abstract

**Background:**

An improved understanding of the trajectory of recovery after mild traumatic brain injury is important to be able to understand individual patient outcomes, for longitudinal patient care and to aid the design of clinical trials.

**Objective:**

To explore changes in health, well-being and cognition over the 2 years following mTBI using latent growth curve (LGC) modelling.

**Methods:**

Sixty-one adults with mTBI presenting to a UK Major Trauma Centre completed comprehensive longitudinal assessment at up to five time points after injury: 2 weeks, 3 months, 6 months, 1 year and 2 years.

**Results:**

Persisting problems were seen with neurological symptoms, cognitive issues and poor quality of life measures including 28% reporting incomplete recovery on the Glasgow Outcome Score Extended at 2 years. Harmful drinking, depression, psychological distress, disability, episodic memory and working memory did not improve significantly over the 2 years following injury. For other measures, including the Rivermead Post-Concussion Symptoms and Quality of Life after Brain Injury (QOLIBRI), LGC analysis revealed significant improvement over time with recovery tending to plateau at 3–6 months.

**Interpretation:**

Significant impairment may persist as late as 2 years after mTBI despite some recovery over time. Longitudinal analyses which make use of all available data indicate that recovery from mTBI occurs over a longer timescale than is commonly believed. These findings point to the need for long-term management of mTBI targeting individuals with persisting impairment.

**Electronic supplementary material:**

The online version of this article (10.1007/s00415-020-09979-x) contains supplementary material, which is available to authorized users.

## Introduction

It is estimated that up to 50 million people sustain a traumatic brain injury (TBI) worldwide every year [30]. Of these, over 90 per cent may be classified as “mild” based on the patient’s level of consciousness at presentation [52]. Such classification, however, is a misnomer as many of these patients are left with long-term adverse sequelae and disability. Despite the significant public health burden, our understanding of the trajectory of long-term recovery from mild TBI (mTBI) is limited. An improved understanding of the long-term sequelae after mTBI is important for ongoing clinical care of such patients, especially given the ongoing controversy over whether all mTBI patients recover within weeks versus a significant proportion having persisting ongoing disabling symptoms for months to years after the event [4, 37, 38].

While traditionally defined an initial Glasgow Coma Score of 13–15, loss of consciousness of less than 30 min and post traumatic amnesia no greater than 24 h the clinical spectrum of mTBI is broad, encompassing patients with subtle clinical signs and normal neuroimaging through those with clear evidence of injury on scans and/or more disabling neurological dysfunction [2, 34, 46]. Clinical outcome in mTBI can range from full and rapid recovery to chronic disabling symptoms [30]. A recent large cohort study found that 53% of patients with mTBI who had presented to US level 1 trauma centres reported continuing functional limitations at 1 year [37, 38]. These persisting symptoms are associated with mental health conditions such as post-traumatic stress disorder (PTSD), depression and suicide [30]. The impact of mTBI on quality of life (QoL) over time has been relatively unstudied [12].

Evidence for the breadth and duration of cognitive impairment following mTBI is mixed. Some studies report impairment spanning a wide range of domains: attention, working memory, episodic memory, information processing speed and general cognition [21], whereas others emphasise deficits in attention and memory [17]. Early resolution of cognitive impairment (after 1 week [55] or 1 month [49]) is suggested by some studies, whereas later resolution (after 3 [17, 23] or 12 [21] months) is suggested by others. Such differences in findings may reflect differing enrolment and severity characteristics.

The primary aim of this study was to examine longitudinal changes after mTBI, over an unusually long follow-up period of 2 years, in a comprehensive range of outcomes encompassing neurocognition, psychiatric symptoms, symptoms associated with post-concussion syndrome and quality of life.

## Materials and methods

### Participants

Seventy-three patients were recruited in the Emergency Department between 2012 and 2013 as part of the Acute Brain Injury Program at Addenbrooke’s Hospital, Cambridge, United Kingdom which is a Major Trauma Center. Inclusion criteria included a Glasgow Coma Scale (GCS) score 13–15 on arrival to the Emergency Department (ED) and eligibility for Computed Tomography (CT) head due to head trauma according to NICE guidelines [1]. Patients with a past history of previous TBI, psychiatric or neurological disorder were excluded. Participants were included in this analysis only if they attended a minimum of one follow-up session (*n* = 61).

An additional 25 adults with extracranial injury only were recruited in the Emergency Department and had follow-up assessments 3 months after injury and 99 healthy adults were also recruited. Control participants with suspected current or previous psychiatric disorder, neurological disorder, previous history of TBI or possible current TBI were excluded.

The Local Research Ethics Committee (NRES Committee Norfolk REC EE 0395) approved the study and written informed consent was obtained from all participants.

### Neuropsychological assessment

Face-to-face assessment of health, quality of life (Qol) and cognitive function were completed at 2 weeks (median = 13 days, range = 6–23), 3 months (median = 92 days, range = 81–129), 6 months (median = 193 days, range = 169–253), 1 year (median = 375 days, range = 357–431) and 2 years (median = 736 days, range = 681–841) after injury. Very rarely (< 2% of all instances) participants took home any questionnaires that were not completed during the face-to-face session due to time constraints and returned them by post. The GOSE and all neurocognitive assessments were always performed face-to-face.

### Health and QoL assessment

A battery of questionnaires was used to assess physical health, mental health and QoL: Alcohol Use Disorders Identification Test (AUDIT) [48] assessed drinking behaviour and alcohol-related problems; Beck Depression Inventory-II (BDI-II) [6] measured the magnitude of cognitive, behavioural and physiological symptoms of depression; PTSD Checklist -Civilian Version (PCL-C) [7] assessed PTSD symptoms; Brief Symptom Inventory-18 (BSI-18) [14] assessed psychological distress indicated by somatisation, depression and anxiety; Quality of Life after Brain Injury (QOLIBRI) [56] assessed TBI-specific QoL; Short Form-36 Health Survey (SF36) [8] assessed health-related QoL; Rivermead Post-Concussion Symptoms Questionnaire (RPQ) [24] assessed neurological/somatic, emotional and cognitive post-concussion symptoms; Glasgow Outcome Scale Extended (GOSE) [58] structured interview categorised functional outcome (level of disability/recovery). All questionnaires have good reliability and validity and have been employed in previous TBI outcome studies [39, 41, 52, 57].

Conventional dichotomisation of outcomes between GOSE 4 (Upper Severe Disability) and GOSE 5 (Lower Moderate Disability) was not appropriate for this cohort of patients, in whom an outcome would need to be back to or close to baseline to be classed as favourable. We, therefore, defined failure to achieve a Good Recovery (GOSE < 8) as an unfavourable outcome. This approach is in keeping with current views on using sliding dichotomy or proportional odds methods in TBI studies [26].

### Cognitive functioning assessment

Cognitive functioning was assessed using classic phonemic (F A S words) and semantic (animal category words) verbal fluency tasks [19] and a selection of computerised tasks from the Cambridge Neuropsychological Test Automated Battery (CANTAB, cambridgecognition.com; administered on a Paceblade tablet). The battery of CANTAB tasks selected comprised the folowing: Motor Screening (MOT; screened for visual, motor and comprehension problems), Paired Associates Learning (PAL; episodic memory), Pattern Recognition Memory (PRM; visual recognition memory), Spatial Recognition Memory (SRM; spatial recognition memory), Spatial Span (SSP; visuospatial working memory capacity), Spatial Working Memory (SWM; retention and manipulation of visuospatial information, strategy use), Rapid Visual Information Processing (RVP; sustained attention), and Intra-Extra Dimensional Set Shift (IED; visual discrimination, attentional flexibility). CANTAB tasks are sensitive to neurocognitive dysfunction following TBI [29, 39–41].

### Statistical analysis

Incidence of various health conditions at each time point was calculated using published cut-off scores; harmful drinking (AUDIT ≥ 8) [11], depression (BDI-II ≥ 14 [6], PTSD (PCL-C ≥ 33) [5], post-concussion syndrome (RPQ ≥ 3) [51] and GOSE < 8 [26].

Our analyses used Latent Growth Curve (LGC) modelling techniques. LGC techniques are based on structural equation modelling and use repeated measures to estimate trajectories [25, 50]. Unlike traditional statistical techniques (e.g., repeated measures analysis of variance), they allow for the inclusion of participants with missing data (e.g., who dropped out or missed a testing session) enhancing the generalisability of findings. Inclusion of all data is particularly important in TBI research due to high attrition rates [45]. In addition to temporal changes at the group level, LGC modelling can also be used to explore individual differences in both temporal changes and initial status. Longitudinal changes in mean outcome scores were examined using LGC modelling in a structural equation modelling environment [25, 50]. The approach has been applied to modelling long-term changes in outcome after rehabilitation for TBI [25]. LGC modelling allowed for an exploration of the initial status of each measure (intercept), inter-individual differences in initial status (variance in intercept) and rate of change (variance in slope), and rate of group change over time (slope) using all available data. In cases where the linear LGC model was significant, further quadratic and cubic models were calculated; maximum likelihood estimation was used to determine which model provided the best fit for the data. The distribution for CANTAB variables deviated from the normal distribution at some time points (as demonstrated by Shapiro–Wilk tests and P–P plots); prior to LGC analysis CANTAB scores were, therefore, transformed using square root transformation to help normalize the multivariate distributions (reverse transformation was first performed on variables for which lower scores equalled better performance). Growth curves were plotted with mTBI group means are plotted against trauma control norms (mean ± 1 SE) for health and QoL variables and against healthy control norms for the CANATB neurocognitive variables. Statistical analysis was performed using IBM SPSS Statistics (Version 25.0; Armonk, NY, USA).

## Results

Table 1 presents participant demographic and injury characteristics. The majority of participants with mTBI (77%) were GCS 15 on arrival, although patients with GCS 13 (5%) and GCS 14 (18%) were also represented. Sixty-nine percent were male, and the age range 17–84 years [mean = 42.0 years, Standard Deviation (SD) ± 17.77]. The estimated premorbid IQ range was 97–124 (mean = 113.72, SD ± 7.22) using the National Adult Reading Test (NART) [36]. The trauma controls were 52% male with an age range 18–59 years (mean = 35.0 years, SD ± 12.42) and 99 healthy adults 49% male with an age range 18 to 70 years (mean = 36.3 years, SD ± 11.99).

**Table 1:** Participant characteristics across the groups of healthy volunteers, trauma controls and patients with mild traumatic brain injury

Participant Characteristic	Healthy volunteer controls (*n* = 99)	Trauma controls (*n* = 25)	mTBI (*n* = 61)	Kruskal–Wallis^a^ or Chi-Square^b^		
				*df*	*H* or *χ*^2^	*p*
Age, mean (SD), years	36 (12)	35 (12)	42 (18)	2	3.2^a^	0.202
Sex, *n* (%) female	50 (51)	12 (48)	19 (31)	2	5.77^b^	0.056
GCS, *n* (%)						
15	–	–	47 (77)			
14			11 (18)			
13			3 (5)			
AIS total, median (range)	–	1 (1–5)	4 (1–16)			
AIS extracranial, median (range)	–	1 (1–5)	2 (1–12)			
Marshall score, *n* (%)						
I	–	–	53 (87)			
II	–	–	7 (11.4)			
III	–	–	0			
IV	–	–	0			
V	–	–	0			
VI	–	–	1 (1.6)			
Mechanism of injury						
Road traffic collision	–	7 (28)	14 (23)	6	15.44^b^	0.017
Fall	–	9 (36)	21 (34)			
Violence or assault	–	0	6 (10)			
Blow to head (not assault)	–	0	7 (12)			
Rugby injury	–	2(8)	4(7)			
Cycling or Horse riding	–	3(12)	9(15)			
Blow to body (not head)	–	4(16)	0			
Education, *n* (%)						
Low	41 (43)	10 (40)	19 (31)	4	2.41^b^	0.660
Middle	26 (27)	5 (20)	17 (28)			
High	29 (30)	10 (40)	25 (41)			

*mTBI* mild traumatic brain injury, *GCS* Glasgow Coma Scale, *AIS* Abbreviated Injury Scale, *SD* standard deviation, *n* number

### Attendance over time

The number of participants in attendance at each follow-up session decreased over time from 56 (92% of the sample) at 2 weeks to 31 (51%) at 2 years (3 months, *n* = 52, 85%; 6 months, *n* = 41, 67%; 1 year, *n* = 34, 56%). Fifty two participants (85% of the sample) attended at least two follow-up sessions and 22 participants (36%) attended all five follow-up sessions (≥ 3 sessions, *n* = 44, 72%; ≥ 4 sessions, *n* = 35, 57%). No patients died during the study period to explain the loss to follow-up. While exact causes of attrition were unable to be obtained the rate described is consistent with previous TBI studies [44].

### Incidence of health conditions and post-concussion symptoms

**Fig. 1:**
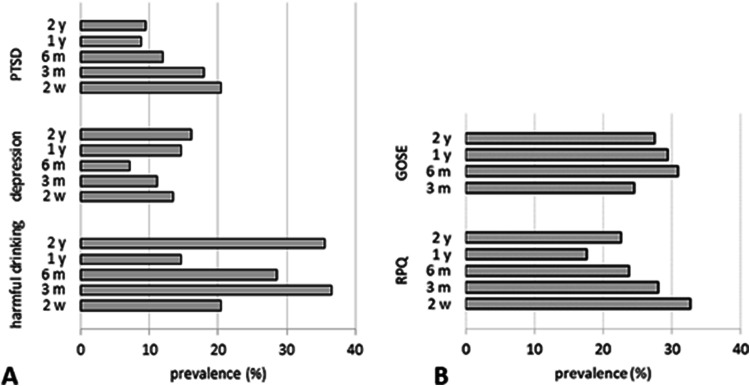
Incidence of health conditions for patients with mild traumatic brain injury—harmful drinking (**a** AUDIT ≥ 8), depression (**a** BDI-II ≥ 14), PTSD (**a** PCL-C ≥ 33), PCS (**b** RPQ ≥ 3), disability (**b** GOSE < 8)—at each time point. *PCS* post-concussion syndrome, *PTSD* post-traumatic stress disorder, *AUDIT* Alcohol Use Disorders Identification Test, *BDI* Beck Depression Inventory, *PCL-C* PTSD Checklist-Civilian Version, *RPQ* Rivermead Post-Concussion Symptoms Questionnaire, *GOSE* Glasgow Outcome Score Extended

**Fig. 2:**
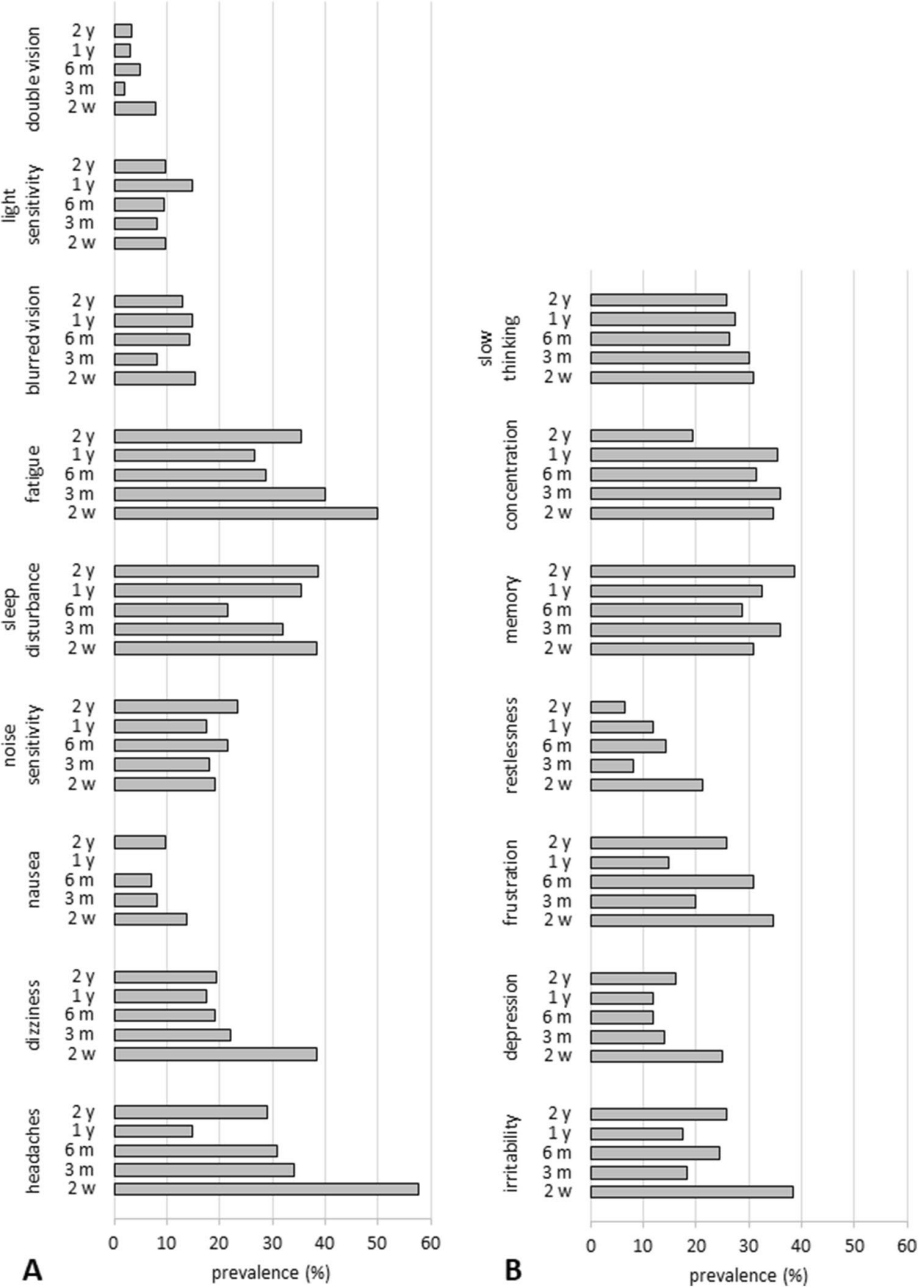
Incidence of self-reported post-concussion symptoms for patients with mild traumatic brain injury—neurological (**a**), emotional (**b**), cognitive (post-concussion symptoms in complicated vs. uncomplicated mild traumatic brain injury patients at three and six months post-injury: results from the CENTER-TBI study)—at each time point

Figure 1 displays incidence rates of harmful drinking, depression, PTSD, post-concussion syndrome and disability, and Fig. 2 displays the percentage of participants reporting specific neurological, emotional and cognitive post-concussion symptoms at each time point (Figs. 1, 2). Incidence of harmful/hazardous drinking was as high at 2 years as it was at 3 months. Incidence of depression decreased steadily up to 6 months and then rose to slightly higher than 2-week levels. Incidences of PTSD, post-concussion syndrome and moderate-to-severe disability each decreased more steadily, but disability was noticeably lowest at 6 months.

Self-reported neurological symptoms tended to be most prevalent at 2 weeks; headaches, fatigue, dizziness and sleep disturbance were reported most frequently. Incidence of headaches and dizziness decreased rapidly up to 3 months and then stayed relatively constant, although incidence of headaches was noticeably lowest at 1 year. Incidence of sleep disturbance decreased steadily up to 6 months then rose to 2-week levels. Incidence of fatigue decreased steadily to 1 year then rose again. Nausea, noise/light sensitivity and blurred/double vision did not fluctuate much over time.

Self-reported emotional symptoms were also most prevalent at 2 weeks with irritability and frustration being more common than depression and restlessness. Incidence of each emotional symptom decreased rapidly up to 3 months. After 3 months, incidence of depression remained relatively stable, whereas incidences of restlessness, irritability and frustration rose again at 6 months.

Self-reported issues with cognition were reported by no less than one-fifth of participants at any time point. Incidences of concentration problems and slowed thinking declined from 2 weeks through 2 years, whereas the opposite pattern was observed for memory problems which increased over time.

### Longitudinal changes in outcome

**Fig. 3:**
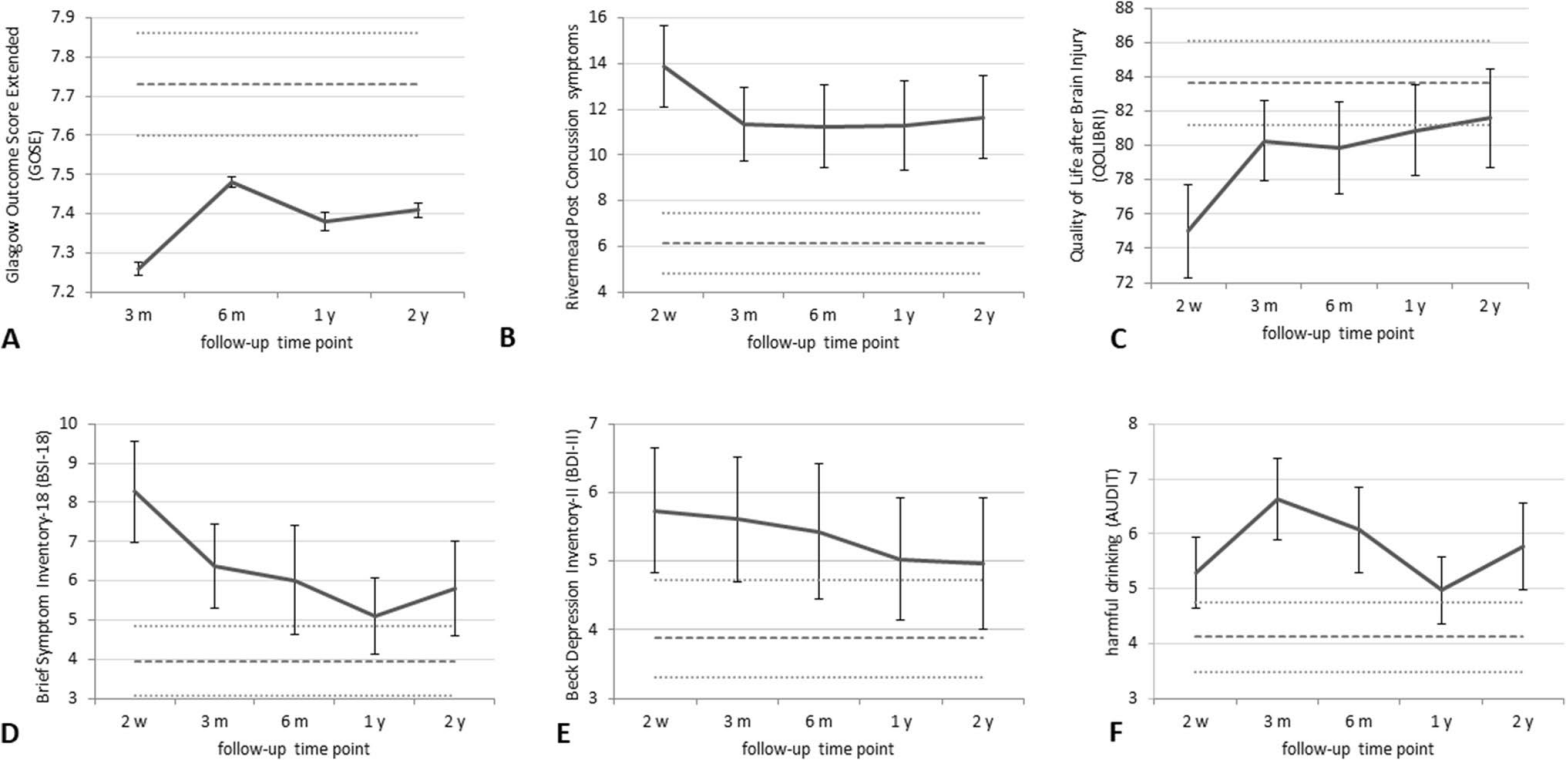
Mean health scores for patients with mild traumatic brain injury: Glasgow Outcome Score Extended (GOSE) (**a**), Rivermead Post-Concussion Questionnaire number of symptoms (RPQ) (**b**), quality of life after brain injury (QOLIBRI) (**c**), psychological distress (Brief Symptom Inventory-18, BSI-18) (**d**), depression [Beck depression inventory-II (BDI-II)] and **e** harmful drinking [Alcohol Use Disorders Identification Test (AUDIT)] (**f**). Error bars represent standard error. Dotted lines represent mean (± 1 SE) scores in trauma controls at 3 months after extracranial injury

The figures showing trajectories (Fig. 3, Supplementary Fig. 1 to 4) show a general trend for improvement over time, although the magnitude and rate of change varied substantially across variables. In the majority of cases, improvement appeared most rapid in the first 3 months after injury for health and QoL variables and in the first 6 months after injury for cognitive variables.

The trajectory of key mean scores to 2 years post injury can be seen in Fig. 3. In the patient cohort, scores on GOSE, BSI-19 and BDI were worse, and harmful drinking significantly more common than control norms at least until 3 months, and remained so for some outcomes at every follow-up time point. Any change in these variables were not significant as demonstrated by non-significant linear LGC model slopes (Table 2). The rate of improvement in QOLIBRI was rapid for the first 3 months and then decelerated with QOLIBRI not reaching trauma control norms until 2 years after injury (Fig. 3). The best fit for this trajectory was quadratic with a significant model slope (*p* = 0.034). The Rivermead post-concussion symptoms appeared to improve rapidly up to 3 months and then plateaued up to 1 year. The cubic LGC model provided the best fit for improvements in post-concussion symptoms demonstrated by a significant cubic LGC slope and significant differences between the − 2LL for the quadratic and cubic models. Detailed results for SF-36 and the CANTAB neurocognitive battery may be found in the Supplementary Material (Supplementary Fig. 1 to 4, Table 2).

**Table 2:** Latent growth curve modelling results for patients with mild traumatic brain injury

Outcome measure	LGC model	Group intercept			Group slope			Variance of intercept		Variance of slope		Covariance of intercept and slope dual		Residual	
		*df*	*F*	*p*	*df*	*F*	*p*	*Z*	*p*	*Z*	*p*	*Z*	*p*	*Z*	*p*
AUDIT	Linear	1, 58.78	101.37	< 0.001	1, 24.78	1.60	0.217	5.00	< 0.001	0.80	0.427	− 1.44	0.151	7.14	< 0.001
DBI-II	Linear	1, 57.62	67.29	< 0.001	1, 36.46	0.92	0.346	3.81	< 0.001	0.39	0.698	− 0.55	0.585	7.62	< 0.001
BSI-18	Linear	1, 50.69	56.35	< 0.001	1, 24.05	3.23	0.085	3.19	0.001	0.29	0.771	− 1.15	0.250	7.18	< 0.001
RPQ	Cubic	1, 75.71	85.69	< 0.001	1, 123.26	4.93	0.028	4.60	< 0.001	1.35	0.176	− 1.99	0.047	7.62	< 0.001
GOSE outcome	Lin	1, 56.54	3576.66	< 0.001	1, 37.23	0.90	0.350	2.12	0.024	0.66	0.507	− 0.41	0.681	6.19	< 0.001
QOLIBRI	Quadratic	1, 68.84	1143.78	< 0.001	1, 130.27	4.58	0.034	4.48	< 0.001	1.55	0.121	− 1.98	0.048	7.30	< 0.001
Physical functioning	Linear	1, 59.54	997.20	< 0.001	1, 36.05	2.68	0.002	4.28	< 0.001	1.61	0.109	− 2.33	0.020	7.24	< 0.001
Social functioning	Cubic	1, 108.80	469.02	< 0.001	1, 123.71	10.65	0.001	3.24	< 0.001	1.08	0.283	− 1.89	0.058	7.53	< 0.001
Role physical	Cubic	1, 86.14	74.94	< 0.001	1, 111.94	6.98	0.009	4.01	< 0.001	1.25	0.212	− 1.90	0.058	7.18	< 0.001
Role emotional	Linear	1, 50.57	531.99	< 0.001	1, 31.18	3.99	0.055	3.19	0.001	0.46	0.645	− 1.45	0.146	7.20	< 0.001
Emotional well-being	Linear	1, 24.92	1518.38	< 0.001	1, 3.43	10.62	0.039	2.17	0.030	–	–	− 0.23	0.821	3.44	0.001
Energy and vitality	Linear	1, 45.38	631.71	< 0.001	1, 101.25	10.48	0.002	2.97	0.003	–	–	− 0.15	0.883	7.58	< 0.001
Bodily pain	Cubic	1, 92.97	243.69	< 0.001	1, 108.79	16.84	< 0.001	3.98	< 0.001	1.37	0.169	− 1.43	0.152	7.03	< 0.001
General health	Linear	1, 56.94	803.64	< 0.001	1, 31.18	0.06	0.805	4.52	< 0.001	0.20	0.840	1.09	0.277	7.36	< 0.001
Phonemic VF	Quadratic	1, 65.24	896.60	< 0.001	1, 129.42	10.92	0.001	4.33	< 0.001	0.32	0.751	0.42	0.672	7.10	< 0.001
Semantic VF	Linear	1, 53.17	1386.64	< 0.001	1, 143.03	4.90	0.028	3.25	0.001	–	–	0.89	0.375	8.06	< 0.001
PAL errors	Linear	1, 68.17	9356.77	< 0.001	1, 156.98	0.28	0.601	3.64	< 0.001	–	–	–	–	8.48	< 0.001
PAL stages completed	Linear	1, 37.76	5239.13	< 0.001	1, 147.42	0.02	0.900	2.60	0.009	–	–	0.43	0.670	8.15	< 0.001
SRM latency	Linear	1, 57.33	4351.57	< 0.001	1, 147.95	1.52	0.219	3.20	0.001	–	–	–	–	8.15	< 0.001
SRM percent correct	Quadratic	1, 86.80	14,508.99	< 0.001	1, 164.66	6.64	0.011	3.46	0.001	–	–	− 0.19	0.854	8.85	< 0.001
PRM latency	Cubic	1, 93.54	4385.64	< 0.001	1, 111.46	6.46	0.012	3.81	< 0.001	1.71	0.088	− 2.90	0.004	7.19	< 0.001
PRM percent correct	Linear	1, 54.43	19,204.29	< 0.001	1, 143.52	2.00	0.160	3.88	< 0.001	–	–	− 0.96	0.338	8.43	< 0.001
SSP span length	Linear	1, 59.13	4174.25	< 0.001	1, 36.12	0.27	0.607	4.54	< 0.001	1.45	0.147	0.26	0.797	7.33	< 0.001
SWM between errors	Linear	1, 60.58	1663.93	< 0.001	1, 0.38	34.52	0.540	4.77	< 0.001	1.65	0.098	− 0.49	0.627	7.37	< 0.001
SWM within errors	Linear	1, 57.38	4865.50	< 0.001	1, 25.85	0.13	0.722	4.08	< 0.001	1.95	0.051	− 2.87	0.004	7.33	< 0.001
SWM strategy	Linear	1, 59.44	787.49	< 0.001	1, 36.21	7.88	0.008	4.77	< 0.001	1.19	0.234	− 0.43	0.664	7.44	< 0.001
RVP A prime	Quadratic	1, 67.20	67,125.62	< 0.001	1, 139.08	6.55	0.012	4.61	< 0.001	–	–	0.92	0.360	8.14	< 0.001
RVP latency	Linear	1, 47.46	2410.03	< 0.001	1, 93.46	4.99	0.028	3.36	0.001	–	–	–	–	6.65	< 0.001
IED pre-ED errors	Quadratic	1, 68.22	999.26	< 0.001	1, 73.72	12.23	0.001	4.09	< 0.001	–	–	–	–	6.28	< 0.001
IED ED errors	Linear	1, 0.01	2890.56	0.944	1, 0.03	2.90	0.917	0.04	0.970	–	–	–	–	0.24	< 0.001
IED reversal errors	Linear	1, 286.05	6861.42	< 0.001	1, 147.47	0.13	0.719	–	–	–	–	–	–	8.13	< 0.001

LGC = latent growth curve; PCS = post-concussion syndrome; GOSE = Glasgow Outcome Score Extended; QoL = quality of life; VF = verbal fluency; PAL = paired associates learning; SRM = spatial recognition memory; PRM = pattern recognition memory; SSP = spatial span; SWM = spatial working memory; RVP = rapid visual information processing; IED = inter-extra dimensional set shifting; ED = extra dimensional

## Discussion

This study examined recovery from mTBI over 2 years and found that ongoing symptoms were common for the entire period. Importantly, it is the first study to report functional, quality of life and neurocognitive outcomes over such a long time period. Although improvement was rapid early on (before plateauing) for post-concussion symptoms (significant improvement up to 3 months) and GOSE, scores fell below trauma control norms at every time point. The overall persistence of symptoms was high: 2 years after injury, one-third of the sample reported headaches, fatigue or sleep disturbances, and one-fifth had limitations with a GOSE < 8. Incidence of unfavourable outcome at earlier time points was similar to figures reported elsewhere [12]. Consistent with prior research, symptoms commonly associated with post-concussion syndrome including headaches, fatigue, dizziness and sleep disturbance were the most common symptoms [43]. Nausea, noise/light sensitivity, and blurred/double vision were less common but did not diminish over time.

Mental health-related symptoms were common: incidences of depression (16%), PTSD (9%) and harmful drinking (35%) were above national averages even 2 years post-injury [15]. Feelings of irritability and frustration fell from 40% at 2 weeks to 15% at 2 years. Incidence of PTSD following mTBI was similar to that reported elsewhere, whereas incidence of depression was higher here [20]. Depression and sleep disturbance followed a similar pattern of steady decrease in incidence up to 6 months before rising to 2 week levels, suggesting that the “neurological” symptoms of the RPQ may be a consequence of depression [22]. Depression is one of the most common psychiatric sequelae in survivors of TBI, and its presence is associated with impaired functional and cognitive recovery and increased disability [3]. The exact mechanisms are unclear but are thought to be (at least in part) secondary to altered functional connectivity of networks associated with emotional regulation [35].

Depression, psychological distress and harmful drinking scores were poorer than trauma control norms at all time points (although longitudinal changes were not significant). Interestingly, the two measures of depression revealed different 2-week incidence rates: reporting depressed mood on the RPQ (25%) was more common than being classified as depressed on the BDI-II (14%). The act of reporting other symptoms during completion of the RPQ may have enhanced perception of current low mood highlighting the importance of considering contextual factors when analysing questionnaire data [54].

Ongoing lowered QoL was predominately due to emotional rather than physical health factors. Physical-related, social-related and TBI-specific QoL improved rapidly early-on and tended to reach trauma control norms by 3 months. Bodily pain scores were better than trauma control norms, which is unsurprising given the nature of their extracranial injuries. Emotional-related QoL improved steadily through all time points, and like TBI-specific QoL (which combined emotional, physical, social and cognitive factors), did not reach trauma control norms until 1 or 2 year(s). Temporal changes in QoL after mTBI have been little studied, although links between emotional health (depression and PTSD) following mTBI and lowered QoL have been reported [20].

Cognitive problems—slowed thinking, poor concentration or memory problems—were reported by no less than one-fifth of participants at any given time point. Self-reports were corroborated by actual performance on the cognitive tasks in almost every case. Concentration problems were reported by approximately one-third of the sample up to 1 year before dropping considerably; this was mirrored by performance on the sustained attention task on which improvement (RVP A prime) was slow up to 1 year and then accelerated. Self-reports of slowed thinking decreased over time (from 31 to 26%) with a slight increase between 6 months and 1 year; this was reflected in latency scores which improved steadily over time (RVP latency) or improved rapidly up to 6 months before worsening (PRM latency). Memory problems were reported more often as time progressed and by more than approximately one-third of the sample at all time points; similarly, performance on the memory tasks tended to remain lower than healthy control norms. Consistent with other literature, memory issues were more common than poor concentration and slowed thinking [28], and reductions in response speed [32] and attention deficits [31] following mTBI are particularly common in the first few months after injury but can continue to 1 year or later [42].

Temporal changes were also observed for the majority of cognitive domains (all except working memory). Improvement in semantic verbal fluency was steady over time, whereas improvement in phonemic verbal fluency was rapid up to 6 months then slower. Phonemic verbal fluency was below healthy control norms only at 2 weeks. Greater semantic relative to phonemic verbal fluency deficits in mild-to-moderate TBI have been reported elsewhere [18]. Attentional flexibility and working memory accuracy scores were poorer than healthy control norms at all time points. Attentional flexibility (IED pre-ED errors) improvement was rapid until 3 months then decelerated but working memory improvement was non-significant over time. Counter to the abrupt nature of strategy discovery reported elsewhere [27], strategy use (SWM strategy) improved linearly over time but never reached healthy control norms. Evidence for working memory deficits following mTBI is mixed; working memory deficits have been reported in some studies [13, 55], but other studies have found no performance differences between mTBI patients and controls despite differential brain activation patterns with increased working memory load [33].

Future studies using imaging techniques such as diffusion tensor imaging (DTI), functional magnetic resonance imaging and spectroscopy may provide valuable insight into the causes of prolonged issues in mTBI. For example, DTI characterises the diffusion of water molecules which is influenced by the microstructural organization of tissues, particularly in white matter. Measuring longitudinal changes from DTI may offer insights into structural connectivity changes and their relationship to outcome. Interestingly, the cognitive measures that worsened over time in our sample (memory and processing speed) are also those linked with decreases in white matter integrity over time in moderate-to-severe TBI [40]. Recent studies have linked diffuse axonal injury in the limbic system with PTSD in mTBI [47]. Indeed, many of the cognitive deficits and mental health symptoms experienced following mTBI may be caused by damaged attentional control pathways [16]. For therapies to be targeted effectively and with the greatest gains in outcome, further studies should establish the causal structure of prolonged problems. Targeted sleep therapy, for example, may not only provide direct resolution of sleep disturbances but may also provide indirect relief of co-occurring problems such as those with cognition and emotion. Similarly, targeted therapy for depression may also improve cognitive functioning [10]. In addition, outcome may be influenced by the presence of extra-cranial injury, and larger studies may allow the effects of this to be fully elucidated [9].

In this study attendance rate fell from 92 to 51% between 2 weeks and 2 years after injury and only 36% of our sample of adults with mTBI attended all five testing occasions. By employing LGC modelling instead of more traditional analysis techniques we avoided substantial loss of power, bias and lowered generalisability because data from participants that missed testing sessions could be included.

Limitations to this study included the relatively small sample size and lack of control for practice effects and some premorbid factors. A significant proportion of the variance in all LGC models was left unexplained and the sample size prevented an investigation of predictive variables via the addition of various covariates. Despite efforts to exclude participants with diagnosed psychiatric conditions, premorbid levels of depression, anxiety and substance abuse, which are known predictors of post-TBI functioning [42], were not recorded. It is, therefore, impossible to separate the apparent consequences of mTBI observed here from existing characteristics. Practice effects are an unavoidable consequence of repeated cognitive testing, especially on novel executive tasks [53] and tasks promoting strategy formation [5], and are observed on many of the CANTAB tasks employed here. Unfortunately, normative data, separated by age and ability level, on serial CANTAB testing are not available. However, our inclusion of control norms and self-report measures of cognition help to overcome some of the difficulty with distinguishing genuine improvement from task familiarity. At least some influence of recovery over practice was suggested by performance rarely reaching normal levels, even after multiple testing sessions and considerable recovery time, and by corroboration of performance improvements by participants’ self-reports.

The elevated level and duration of cognitive and emotional issues observed here may be attributable to our use of techniques which do not necessitate the removal of cases with missing data. If the nature of the missing data is not random, such that non-attenders tend to have extreme outcomes limiting their ability or motivation to attend, ongoing difficulties will be underestimated [45].

## Conclusion

This study demonstrates that, for some individuals, recovery from mTBI occurs over a long timescale and is much more protracted than usually believed. In particular, problems with emotional well-being, alcohol use and cognition, as well as symptoms such as headaches and sleep disturbance, are common after mTBI and often long-standing. Further LGC modelling in larger samples and over longer time frames will enable exploration of casual factors whilst providing the power to detect potential individual differences in the rate of change over time. The findings point to the need for long-term management of mTBI targeting subgroups with persisting decreases in cognition, mental health problems and functional limitations.

## Data availability statement

Anonymised data used for the analyses in this study are available from the corresponding author upon reasonable request (ORID ID https://orcid.org/0000-0001-6044-9035).

## Electronic supplementary material

Below is the link to the electronic supplementary material.

Supplementary file1 (DOCX 534 kb)
