# Supplementary material for: Mild traumatic brain injury recovery: a growth curve modelling analysis over 2 years
Source: J Neurol. 2020 Jun 13;267(11):3223–34. doi: 10.1007/s00415-020-09979-x (PMC7578150; doi:10.1007/s00415-020-09979-x)
Supplement: Supplementary file 1 — Supplementary file1 (DOCX 534 kb) [file 415_2020_9979_MOESM1_ESM.docx]

***Supplementary Results: LGC models of health, SF-36 results and neurocognitive functioning***

Table 2 (results) and Supplementary Table 1 (parameters) present the statistics for the LGC models that provide the best fit to the longitudinal data for the SF-36 and CANTAB neurocognitive battery. Growth curves are plotted in Figures 1 to 4; mTBI group means are plotted against trauma control norms (mean ±1 SE) for health and QoL variables and against healthy control norms for cognitive variables.

Some outcome metrics were not well characterised by the range of LGC models explored, whereas others were best described by linear, quadratic, or cubic LGC fits. For every LGC model with a significant slope, both the intercept and the variance in intercept was significant; the initial status of each measure differed significantly from zero and differed significantly across participants.

Any improvements in physical functioning, general health, role emotional, episodic memory, working memory span, working memory maintenance and manipulation, attentional flexibility accuracy in the extra-dimensional and reversal stages, spatial recognition memory speed, and visual recognition memory accuracy were not significant as demonstrated by non-significant linear LGC model slopes. Scores on all of these variables were nonetheless below control norms at least until three or six months and in many cases at all time points (the only exception was role emotional which always fell within trauma control norms).

The linear LGC model provided the best fit for improvements in emotional well-being, energy and vitality, semantic verbal fluency, working memory strategy, and sustained attention speed. This was demonstrated by significant linear LGC slopes, non-significant quadratic LGC slopes, and non-significant differences between the -2 log likelihood (-2LL) for the linear and quadratic models. All of these measures improved steadily between two weeks to two years. Energy and vitality and emotional well-being did not reach trauma control norms until one year and two years, respectively. SWM strategy scores were below healthy control norms until two years, whereas RVP latency was within (or above) normal limits at each time point.

The best fit for improvement, phonemic verbal fluency, spatial recognition memory accuracy (SRM percent correct), sustained attention target sensitivity (RVP A prime), and attentional flexibility accuracy in the pre-extra-dimensional stage (IED pre-errors) was provided by the quadratic LGC model. This was demonstrated by significant quadratic mean slopes and significant differences between the -2LL for the linear and quadratic models. The rate of improvement in phonemic verbal fluency, SRM percent correct and IED pre-ED errors was rapid at first (up to three months; up to six months for phonemic verbal fluency) and then decelerated whereas the rate of RVP A prime improvement was steady except for a plateau between six months and one year. SRM percent correct was lower than in healthy controls at all time points whereas RVP A prime was within (or above) normal limits at every time point. Phonemic verbal fluency and IED pre-ED errors reached normal limits by three months.

The cubic LGC model provided the best fit for improvements in role physical, bodily pain, social functioning, and visual recognition memory speed. This was demonstrated by significant cubic LGC slopes and significant differences between the -2LL for the quadratic and cubic models. The SF-36 variables improved rapidly up to three months; role physical showed improvement then slowed before accelerating again after six months, for bodily pain improvement stayed constant until one year at which point it accelerated slightly, and for social functioning improvement decelerated from three months and decelerated further from six months. PRM latency improved rapidly up to six months at which point it worsened slowly up to one year and then slightly more rapidly. Role physical, social functioning, bodily pain, and PRM latency reached control norms at three months.

For every LGC model with a significant slope, both the intercept and the variance in intercept was significant; the initial status of each measure differed significantly from zero and differed significantly across participants. The variance in slope was not significant for any measure. The covariance between initial status and rate of improvement of was significant for PRM latency; individuals with better initial PRM latency scores exhibited faster improvement in PRM latency over time.


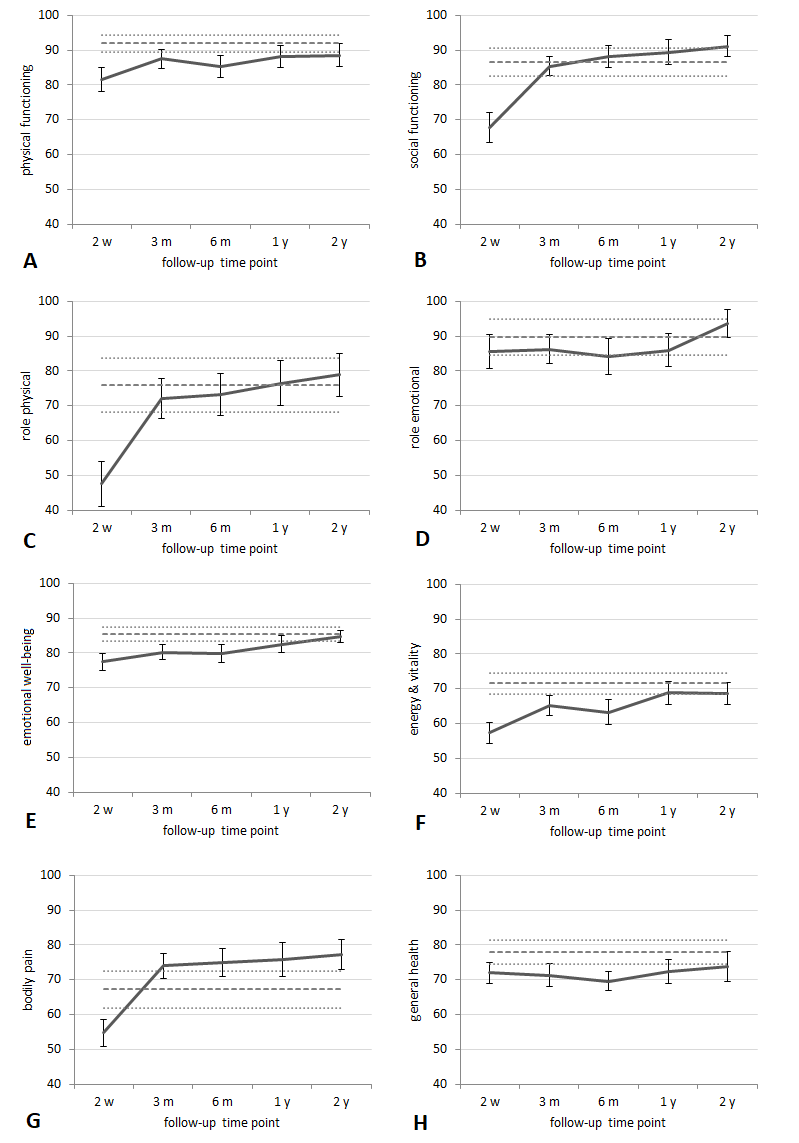


**Supplementary Figure 1.** Mean quality of life scores from SF-36; physical functioning (A), social functioning (B), role physical (C), role emotional (D), emotional well-being (E), energy & vitality (F), bodily pain (G), general health (H)—across follow up time points. Error bars represent standard error. Dotted lines represent mean (±1 SE) scores in trauma controls at three months after extracranial injury.

**
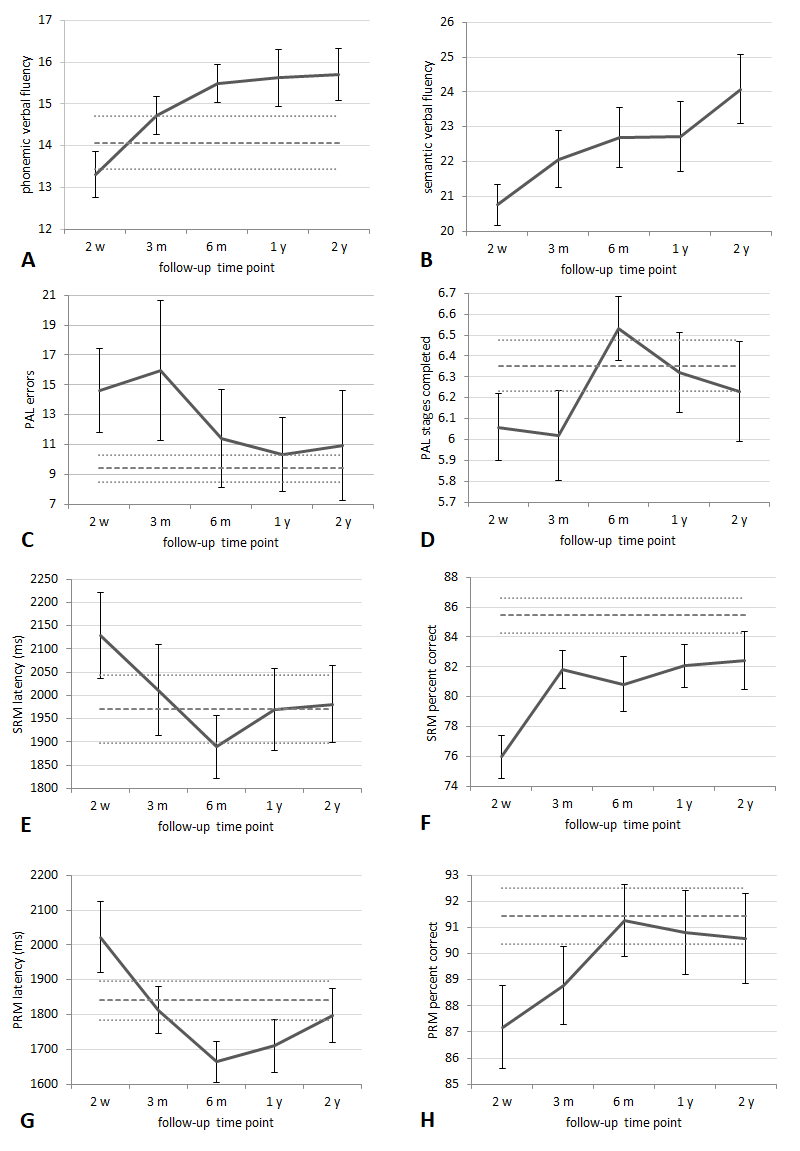
**

**Supplementary Figure 2.** Mean verbal fluency and memory scores from the CANTAB battery of neurocognitive tests; phonemic verbal fluency (A), semantic verbal fluency (B), episodic memory (PAL errors, C; PAL stages completed, D), spatial recognition memory (SRM latency, E; SRM percent correct, F), visual recognition memory (PRM latency, G; PRM percent correct, H)—across follow up time points. PAL errors are adjusted to control for some participants having less opportunity for error due to early termination of the task resulting from poor performance. Error bars represent standard error. Dotted lines represent mean (±1 SE) scores in healthy controls. Abbreviations: PAL = paired associates learning; SRM = spatial recognition memory; PRM = pattern recognition memory.


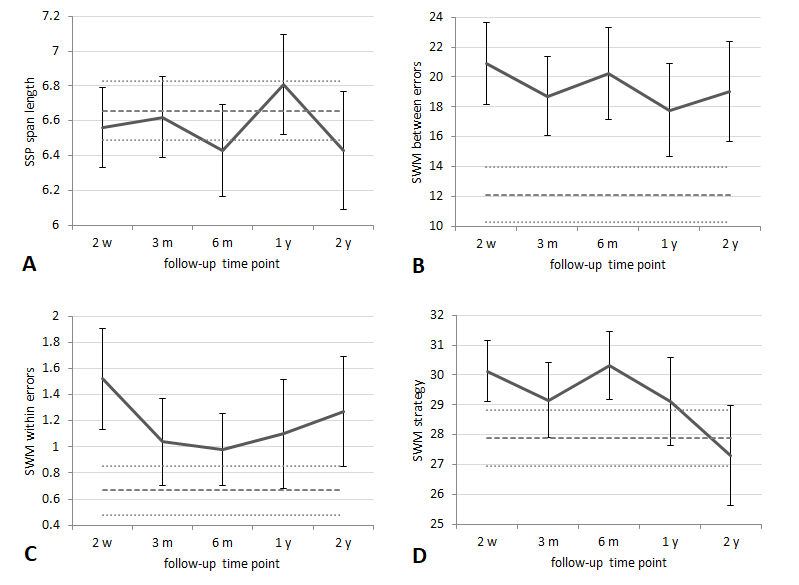


**Supplementary Figure 3.** Mean working memory scores from the CANTAB battery of neurocognitive tests; working memory span (SSP span, A), working memory maintenance and manipulation (SWM between errors, B; SWM within errors, C), strategy use (SWM strategy, D)—across follow up time points. Error bars represent standard error. Dotted lines represent mean (±1 SE) scores in healthy controls. Abbreviations: SSP = spatial span; SWM = spatial working memory.


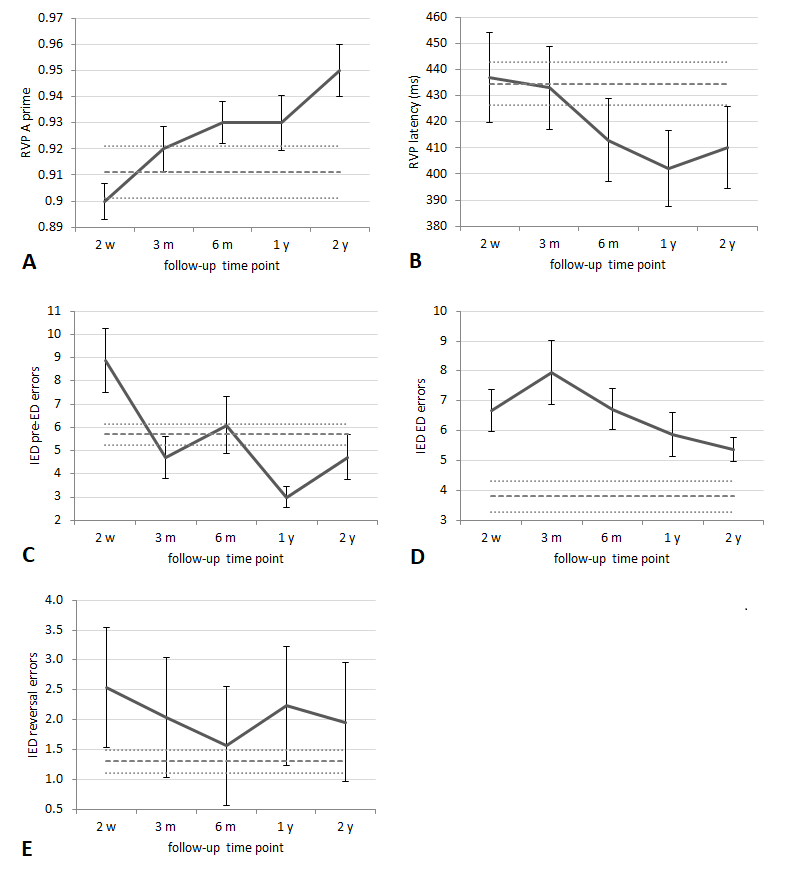
 **Supplementary Figure 4.** Mean attention scores from the CANTAB battery of neurocognitive tests; sustained attention (RVP A prime, A; RVP latency, B), attentional flexibility (IED pre-ED errors, C; IED ED errors, D; IED reversal errors, E)—across follow up time points. Error bars represent standard error. Dotted lines represent mean (±1 SE) scores in healthy controls. Abbreviations: RVP = rapid visual information processing; IED = inter-extra dimensional set shifting; ED = extra dimensional.

**Supplementary Table 1.** Latent growth curve model parameters

| *Outcome measure* | *complete data [n(% sample)]* | *LGC model with best fit* | *LGC model parameters [estimate(SE)]* | | | |
| --- | --- | --- | --- | --- | --- | --- |
|  |  |  | *intercept* | *slope* | *variance of intercept* | *variance of slope* |
| AUDIT | 20 (33) | none | 6.10 (0.61) | -0.54 (0.43) | 20.38 (4.07) | 1.91 (2.40) |
| BDI-II | 20 (33) | none | 5.84 (0.71) | -0.97 (0.91) | 21.79 (5.72) | 3.19 (8.20) |
| BSI-18 | 21 (34) | none | 7.50 (1.00) | -2.44 (1.36) | 38.62 (12.09) | 7.31 (25.08) |
| RPQ | 22 (36) | cubic | 14.38 (1.55) | -50.78 (22.86) | 106.12 (23.08) | 29.36 (21.68) |
| GOSE | 22 (36) | none | 7.33 (0.12) | 0.19 (0.20) | 0.34 (0.16) | 0.27 (0.41) |
| QOLIBRI | 18 (30) | quadratic | 76.12 (2.25) | -15.05 (7.04) | 233.00 (52.07) | 99.92 (64.49) |
| Physical functioning | 20 (33) | none | 83.85 (2.66) | 5.67 (3.46) | 322.32 (75.36) | 207.34 (129.21) |
| Social functioning | 21 (34) | cubic | 68.63 (3.17) | 224.56 (68.80) | 242.71 (74.87) | 202.35 (188.31) |
| Role physical | 20 (33) | cubic | 48.40 (5.59) | 264.06 (99.92) | 1103.94 (275.27) | 632.50 (506.59) |
| Role emotional | 20 (33) | none | 84.31 (3.66) | 10.08 (5.04) | 500.04 (156.81) | 140.95 (305.85) |
| Emotional well-being | 21 (34) | linear | 78.04 (2.00) | 7.78 (2.39) | 167.53 (77.06) | 23.84 (0.00) |
| Energy & vitality | 21 (34) | linear | 60.85 (2.42) | 10.83 (3.35) | 208.83 (70.30) | 0.46 (0.00) |
| Bodily pain | 20 (33) | cubic | 54.70 (3.50) | 263.53 (64.23) | 405.55 (102.00) | 347.96 (253.19) |
| General health | 21 (34) | none | 70.46 (2.49) | 0.56 (2.26) | 299.63 (66.23) | 10.54 (52.35) |
| Phonemic VF | 14 (23) | quadratic | 13.51 (0.45) | -4.75 (1.44) | 9.04 (2.09) | 0.61 (1.93) |
| Semantic VF | 14 (23) | linear | 21.41 (0.58) | 1.93 (0.87) | 12.08 (3.72) | 1.10 (0.00) |
| PAL errors | 21 (34) | none | 14.20 (0.15) | 0.11 (0.20) | 0.81 (0.22) | 0.0002 (0.00) |
| PAL stages completed | 21 (34) | none | 2.46 (0.03) | 0.01 (0.05) | 0.04 (0.02) | 0.01 (0.00) |
| SRM latency | 21 (34) | none | 53.97 (0.82) | 1.45 (1.18) | 23.46 (7.34) | 1.58 (0.00) |
| SRM percent correct | 21 (34) | quadratic | 8.81 (0.07) | -0.84 (0.33) | 0.16 (0.05) | 0.0003 (0.00) |
| PRM latency | 21 (34) | cubic | 58.16 (0.88) | 43.44 (17.09) | 24.44 (6.41) | 20.67 (12.11) |
| PRM percent correct | 21 (34) | none | 9.41 (0.07) | 0.11 (0.08) | 0.20 (0.05) | 0.01 (0.00) |
| SSP span length | 19 (31) | none | 2.55 (0.04) | -0.02 (0.05) | 0.08 (0.02) | 0.03 (0.02) |
| SWM between errors | 18 (30) | none | 7.43 (0.18) | 0.12 (0.20) | 1.70 (0.36) | 0.65 (0.39) |
| SWM within errors | 18 (30) | none | 3.94 (0.06) | 0.03 (0.09) | 0.14 (0.04) | 0.23 (0.12) |
| SWM strategy | 18 (30) | linear | 3.72 (0.13) | 0.36 (0.13) | 0.91 (0.19) | 0.18 (0.15) |
| RVP A prime | 19 (31) | quadratic | 0.95 (0.003) | -0.03 (0.01) | 0.0006 (0.0001) | 0.00003 (0.00) |
| RVP latency | 19 (31) | linear | 20.83 (0.42) | 1.36 (0.61) | 6.95 (2.07) | 1.50 (0.00) |
| IED pre-ED errors | 15 (25) | quadratic | 4.67 (0.15) | -2.15 (0.61) | 0.77 (0.19) | 0.61 (0.00) |
| IED ED errors | 15 (25) | none | 5.22 (0.10) | 0.26 (0.15) | 0.33 (7.58) | 0.18 (0.00) |
| IED reversal errors | 15 (25) | none | 5.07 (0.06) | 0.04 (0.12) | 0.07 (0.00) | 0.04 (0.00) |

For measures with non-significant LGC models, parameters for linear models are shown. Abbreviations: LGC = latent growth curve; AUDIT = Alcohol Use Disorders Identification Test, BDI-II = Beck depression inventory-II, BSI-18 = Brief Symptom Inventory-18, RPQ = Rivermead post-concussion score, GOSE = Glasgow Outcome Score Extended; QOLIBRI = Quality of life after brain injury; VF = verbal fluency; PAL = paired associates learning; SRM = spatial recognition memory; PRM = pattern recognition memory; SSP = spatial span; SWM = spatial working memory; RVP = rapid visual information processing; IED = inter-extra dimensional set shifting; ED = extra dimensional.

**Supplemental Table 2.** Transformed CANTAB data used for LGC model statistics

| *Outcome measure* | *2 week* | | *3 month* | | *6 month* | | *1 year* | | *2 years* | |
| --- | --- | --- | --- | --- | --- | --- | --- | --- | --- | --- |
|  | *M* | *SD* | *M* | *SD* | *M* | *SD* | *M* | *SD* | *M* | *SD* |
| PAL errors | 14.24 | 0.78 | 14.06 | 2.10 | 14.35 | 0.82 | 14.40 | 0.53 | 14.37 | 0.82 |
| PAL stages completed | 2.45 | 0.24 | 2.41 | 0.44 | 2.55 | 0.19 | 2.50 | 0.23 | 2.48 | 0.28 |
| SRM latency | 53.16 | 7.09 | 54.00 | 9.44 | 55.77 | 3.90 | 54.97 | 4.83 | 54.91 | 4.33 |
| SRM percent correct | 8.69 | 0.65 | 9.03 | 0.52 | 8.97 | 0.68 | 9.05 | 0.47 | 9.06 | 0.62 |
| PRM latency | 58.49 | 9.64 | 60.93 | 4.30 | 62.21 | 3.08 | 61.81 | 3.72 | 61.11 | 3.51 |
| PRM percent correct | 9.34 | 0.62 | 9.40 | 0.59 | 9.54 | 0.47 | 9.52 | 0.50 | 9.50 | 0.53 |
| SSP span length | 2.54 | 0.34 | 2.55 | 0.33 | 2.51 | 0.33 | 2.59 | 0.31 | 2.51 | 0.37 |
| SWM between errors | 7.27 | 1.71 | 7.51 | 1.38 | 7.39 | 1.50 | 7.60 | 1.24 | 7.42 | 1.73 |
| SWM within errors | 3.89 | 0.60 | 3.99 | 0.33 | 4.00 | 0.23 | 3.97 | 0.35 | 3.95 | 0.34 |
| SWM strategy | 3.70 | 1.01 | 3.82 | 1.13 | 3.69 | 1.03 | 3.80 | 1.24 | 4.06 | 1.13 |
| RVP A prime | 0.95 | 0.03 | 0.96 | 0.03 | 0.97 | 0.03 | 0.97 | 0.03 | 0.97 | 0.03 |
| RVP latency | 20.59 | 4.32 | 20.89 | 3.19 | 21.44 | 2.66 | 21.78 | 2.02 | 21.62 | 1.96 |
| IED pre-ED errors | 4.60 | 1.43 | 5.17 | 0.80 | 5.00 | 0.96 | 5.38 | 0.25 | 5.20 | 0.49 |
| IED ED errors | 5.31 | 0.63 | 5.08 | 1.14 | 5.30 | 0.44 | 5.38 | 0.45 | 5.44 | 0.19 |
| IED reversal errors | 5.02 | 0.85 | 5.08 | 0.40 | 5.14 | 0.18 | 5.06 | 0.46 | 5.10 | 0.23 |

Abbreviations: LGC = latent growth curve; PAL = paired associates learning; SRM = spatial recognition memory; PRM = pattern recognition memory; SSP = spatial span; SWM = spatial working memory; RVP = rapid visual information processing; IED = inter-extra dimensional set shifting; ED = extra dimensional.
